# Supplementary material for: EBV INFECTION OUTCOMES DETERMINED BY MONOCYTE AND TREG-DRIVEN IMMUNE DYNAMICS IN AN EX VIVO PBMC MODEL
Source: bioRxiv. 2025 Nov 21:2025.11.20.689441. Preprint. [Version 1] doi: 10.1101/2025.11.20.689441 (PMC12667846; doi:10.1101/2025.11.20.689441)
Supplement: 1 [file NIHPP2025.11.20.689441v1-supplement-1.pdf]

bioRxiv preprint doi: https://doi.org/10.1101/2025.11.20.689441; this version posted November 21, 2025. The copyright holder for this preprint (which was not certified by peer review) is the author/funder, who has granted bioRxiv a license to display the preprint in perpetuity. It is made available under aCC-BY 4.0 International license.

Supplementary Data

Supplementary Table S1. Donor demographic and experimental metadata.

Summary of donor characteristics used in EBV infection studies. The table includes donor ID, age, sex, race, and health status, along with experimental annotations indicating whether lymphoblastoid cell lines (LCLs) were successfully generated (Made, Failed, or M/F for mixed outcomes). Columns also denote availability of RNA-seq and flow cytometry data, as well as EBV serostatus.

Supplementary Table S2. Summary of LCL outgrowth efficiency across donors and infection conditions.

Donor PBMCs were infected with EBV at varying multiplicities of infection (MOIs) ranging from 0.1 to 10, and LCL outgrowth was assessed across 8–11 independent trials. Each cell represents the outcome of a single trial under the indicated condition. Donors highlighted in green successfully formed LCLs (LCL Made), while those in purple failed to establish LCLs (LCL Failed) under cyclosporin A–untreated conditions. “o” indicates successful LCL outgrowth; “x” indicates failure. Gray-shaded columns represent MOI conditions not tested for the corresponding donor.

Supplementary Figure S1. Success rate of LCL outgrowth per donor.

Bar plot shows the percentage of successful LCL outgrowths per donor under untreated (green) and Cyclosporin A–treated (gray) conditions. Each bar represents the proportion of successful EBV infections out of total attempts across multiple MOI conditions (see Supplementary Table 2).

Supplementary Figure S2. Flow cytometry gating strategy for major immune cell subsets.

Cells were gated sequentially for lymphocyte population (FSC-A vs. SSC-A), single cells (FSC-A vs. FSC-H), and live cells (Aqua green Live/Dead dye). Lineage-specific markers were then

bioRxiv preprint doi: https://doi.org/10.1101/2025.11.20.689441; this version posted November 21, 2025. The copyright holder for this preprint (which was not certified by peer review) is the author/funder, who has granted bioRxiv a license to display the preprint in perpetuity. It is made available under aCC-BY 4.0 International license.

applied within the live, singlet gate to define major immune subsets, including monocytes, T cells, NK cells, and B cells.

Supplementary Figure S3. Gating strategy for identification of regulatory T cells (Tregs).

Flow cytometry plots showing sequential gating to identify $CD4^{+}FOXP3^{+}CD25^{+}$ regulatory T cells.

Supplementary Figure S4. Consistent upregulation of HIF1A and KLRF1 following EBV infection.

(A) Expression of HIF1A and (B) KLRF1 in PBMCs following *ex vivo* EBV infection, compared to mock-treated controls, across all timepoints (Days 1, 7, and 14). Boxplots show log2-transformed normalized gene counts. p-values were calculated using a paired Student's t-test.

Supplementary Figure S5. LIPA expression and potential mechanism of LIPA in EBV-infected cells.

(A) Flow cytometry plots showing LIPA expression in $CD19^{+}$ B cells (left), $CD3^{+}$ T cells (middle), and $CD14^{+}$ monocytes (right). (B) Relative LIPA mRNA expression measured by qPCR in PBMC infected with EBV compared to mock at Day1. Expression normalized to GUSB. ***$p < 0.001$ by paired t-test.

Supplementary Figure S6. Unsupervised clustering of differentially expressed genes at Day 14.

Heatmap showing hierarchical clustering of over 600 differentially expressed genes (rows) at Day 14, comparing donors in which LCLs were successfully established (green) or failed (purple). Scaled expression values are shown (Z-scores per gene). Clustering was performed using Euclidean distance and complete linkage.

bioRxiv preprint doi: https://doi.org/10.1101/2025.11.20.689441; this version posted November 21, 2025. The copyright holder for this preprint (which was not certified by peer review) is the author/funder, who has granted bioRxiv a license to display the preprint in perpetuity. It is made available under aCC-BY 4.0 International license.

Supplementary Figure S7. Key immune surveillance and ROS-related genes show distinct expression in LCL made versus failed donors.

(A) Heatmap of selected differentially expressed genes (rows) related to immune activation and redox signaling, comparing LCL made (green) and failed (purple) donor groups at Day 14. Expression values are scaled by gene (Z-score). (B-K) Boxplots showing log-transformed normalized expression values of representative genes in EBV-infected and mock conditions for each group.

Supplementary Figure S8. Differential expression of T cell exhaustion-related genes in EBV-infected PBMCs from LCL-failed and LCL-made donors.

Boxplots display $\log_2$ normalized expression values (counts +1) for each gene in peripheral blood mononuclear cells (PBMCs), stratified by donor LCL outcome (Failed vs Made) and infection condition (Mock [blue] vs EBV [red]). (A) ENTPD1, (B) HAVCR2, (C) TOX, (D) SLAMF6.p-values indicate results of unpaired two-tailed Student's t-tests between EBV and mock conditions within each donor group.

Supplementary Figure S9. Treg Gating Strategy and Depletion by RG6292 Treatment

(A–C) Flow cytometry gating strategy: lymphocytes were first identified by forward and side scatter (A), followed by singlet discrimination (B), and viability gating using Aqua green live/dead stain (C). (D) $CD4^+$ and $CD8^+$ T cells were gated from live lymphocytes. (E–F) Representative plots showing frequency of $CD25^+FOXP3^+$ Tregs within the $CD4^+$ compartment in mock-IgG1 treated (E) and RG6292-treated (F) PBMCs. RG6292 selectively depleted $CD4^+CD25^+FOXP3^+$ Tregs. (G–H) gp350 expression on $CD19^+$ B cells at Day 7 post-infection in IgG + EBV-infected (G) or RG6292-treated EBV infected (H) cultures. (I–J) Single-cell RNA-seq violin plots showing

bioRxiv preprint doi: https://doi.org/10.1101/2025.11.20.689441; this version posted November 21, 2025. The copyright holder for this preprint (which was not certified by peer review) is the author/funder, who has granted bioRxiv a license to display the preprint in perpetuity. It is made available under aCC-BY 4.0 International license.

IL2RA expression in Treg clusters from LCL-made (I) and LCL-failed (J) donors under mock and EBV conditions.

Supplementary Figure S10. Enhanced EBV transcript detection with probe enrichment-Seq.

(A) Heatmap of normalized EBV counts (log_2 scale) across Day 0, Day 1, and Day 7 post-infection samples. (B) Line plot comparing log_2(% EBV reads) in samples with and without EBV probe enrichment across multiple donors and time points. (C) Boxplot of log_2(% EBV reads) showing a statistically significant increase in EBV reads in the with probe condition compared to without probe (paired Student's t-test, p = 4.3e-09).
